# Chromatographic Fingerprinting Based on Column Switching Technology for Quality Evaluation of Tianmeng Oral Liquid

**DOI:** 10.1155/2021/2514762

**Published:** 2021-09-29

**Authors:** Meng Yuan, Xiaoyan Liu, Yue Sun, Linlin Wang, Pian Jin, Xiaoqi Zhuang, Hengchang Zang, Zhonghu Zhang, Lei Nie

**Affiliations:** ^1^School of Pharmaceutical Sciences, Cheeloo College of Medicine, Shandong University, Jinan 250012, China; ^2^National Glycoengineering Research Center, Jinan 250012, China; ^3^National Medical Products Administration Key Laboratory for Technology Research and Evaluation of Drug Products, Shandong University, Jinan 250012, China; ^4^Shandong Institute for Food and Drug Control, Jinan 250100, China

## Abstract

Separation power was limited when the conventional high-performance liquid chromatography (HPLC) fingerprinting method based on a single column was used to analyze very complex traditional Chinese medicine (TCM) preparations. In this research, a novel HPLC fingerprinting method based on column switching technology by using a single pump was established for evaluating the quality of Tianmeng oral liquid (TMOL). Twelve batches of TMOL samples were used for constructing HPLC fingerprints. Compared with the 16 common peaks in fingerprinting with a single column, 25 common peaks were achieved with two columns connected through a six-way valve. The similarity analysis combined with bootstrap method was applied to determine the similarity threshold, which was 0.992 to distinguish expired samples and unexpired samples. Principal component analysis (PCA) and hierarchical clustering analysis (HCA) were also applied to classify the TMOL samples, and results revealed that expired and unexpired samples are classified into two categories. The HPLC fingerprinting based on column switching technology with better separation power and higher peak capacity could characterize chemical composition information more comprehensively, providing an effective and alternative method to control and evaluate the quality of TMOL, which would offer a valuable reference for other TCM preparations.

## 1. Introduction

Tianmeng oral liquid (TMOL) is a traditional Chinese medicine (TCM) formula preparation composed of seventeen Chinese herbs following the principles of “Jun (emperor)-Chen (minister)-Zuo (adjuvant)-Shi (courier),” which has the effect of replenishing Qi and tonifying kidney, nourishing heart, and calming mind, and commonly used in the treatment of insomnia caused by deficiency of spleen and kidney [1, 2]. Jun (emperor) includes *Acanthopanax senticosus* and *Polygonatum sibiricum*. Chen (minister) includes *Rehmannia glutinosa*, *Lycium chinense*, *Fructus Mori*, *Epimedium brevicornu*, *Silkworm moth*, *Astragalus membranaceus*, and *Codonopsis pilosula*. Zuo (adjuvant) includes *Wolfiporia cocos*, *Pericarpium Citri Reticulatae*, *Rhizoma Pinelliae*, and *Crataegus pinnatifida*. Shi (courier) is *Strychnos nux-vomica*. For the complex system of TCM, the establishment of its fingerprint is an effective method to evaluate the interbatch consistency and stability of quality and has been internationally recognized [3–5]. TMOL is a compound preparation of TCM with the characteristics of multicomponents and multitargets, and its fingerprint has been established on a single column by high-performance liquid chromatography (HPLC) [6]. In addition, other relevant analytical methods available in the literature for quality evaluation of Tianmeng capsules are primarily based on quantification of a single bioactive component, which is difficult to reflect the quality comprehensively [7–9]. The more chromatographic peaks obtained in HPLC fingerprinting could reflect the completer information about chemical components, which is more conducive to reflect the inner quality of TCM comprehensively [10–12]. However, most researches on fingerprinting are based on a single column and the corresponding fingerprint has limited selectivity and separation power to analyze complex TCM preparations [13–15].

The column switching technique is used to connect chromatographic columns with different stationary phases, and the selectivity and resolution of chromatography could be improved to obtain more chromatographic peaks characterizing more chemical information, which is already widely applied to analyze complex systems [16–18]. Usually, the mode of column switching can be divided into two-column double-pump system, three-column three-pump system, and complex coupling system [19], while more pumps require more hardware costs. To carry out automatic column switching, the instrument should be equipped with a double-pump infusion system and a high-pressure flow path selection valve at least [20, 21]. There are few studies on HPLC fingerprinting methods based on column switching of a single pump to obtain more chromatographic peaks for TCM. Given the existing shortcomings, we proposed to establish a single-pump double-column HPLC column switching system by connecting different polarity columns with a six-way valve, which fully combined the advantages of separation selectivity of different polarity chromatographic columns to effectively increase peak capacity, improve the separation effect of characteristic components, and consequently reflect more comprehensive information in fingerprint.

In this study, the column switching technology based on a single pump was employed for the development of the HPLC fingerprinting method to carry out the quality evaluation of TMOL. The present method was compared with a single-column method in different aspects. Various chemometric methods such as similarity analysis, principal component analysis (PCA), and hierarchical clustering analysis (HCA) were all applied to distinguish different batches of TMOL samples for quality evaluation.

## 2. Materials and Methods

### 2.1. Materials and Reagents

Syringin (purity: >98%) and calycosin 7-O-glucoside (purity: >98%) were purchased from Ruifensi Biotechnology Co., Ltd. (Chengdu, China). Icariin (purity: >98%), hesperidin (purity: >98%), and chlorogenic acid (purity: >98%) were purchased from Mansite Biotechnology Co., Ltd. (Chengdu, China). HPLC grade acetonitrile and methanol were obtained from Tedia (Fairfield, USA) and all other solvents used, including acetic acid, phosphoric acid, and formic acid were analytical grade, were purchased from Sinopharm Chemical Reagent (Beijing, China). Deionized water was purified with a Milli-Q water purification system (Molsheim, France). TMOL samples were produced by Rongchang Pharmaceutical Co., Ltd. (Yantai, China) and were purchased from retail pharmacies. The batch numbers (S1–S12) were 170202, 170204, 170706, 171112, 171121013, 171121021, 171218, 180101, 180103, 180104, 180117, and 180203, respectively.

### 2.2. Column Switching Technology

**Figure 1:**
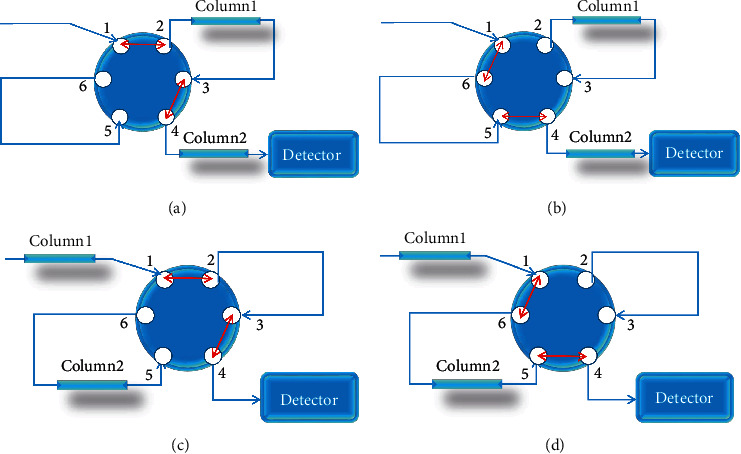
Chromatographic column-connection method of column switching technology based on a single pump.

The HPLC column switching system with a single-pump double-column system was successfully established by adding a six-way injection valve. The two chromatographic columns were connected through a six-way valve. The first kind of connection method is shown in Figures 1(a) and 1(b). The position of the six-way valve in the first 35 min is 1 ⟶ 2 and the two-column analysis was performed. After 35 min, the six-way valve was switched to 1 ⟶ 6 and the single-column analysis was performed. The second kind of connection method is shown in Figures 1(c) and 1(d). The position of the six-way valve in the first 35 min is 1 ⟶ 2 for single-column analysis. The six-way valve was switched to 1 ⟶ 6 after 35 min, and two-column analysis was performed. Samples were injected twice for one sample analysis according to two connection methods, and we focused on the comparison of the single-column method and the two-column method based on column switching technology.

### 2.3. Apparatus and Chromatographic Conditions

Chromatographic separations were carried out by an Agilent 1260 HPLC instrument (Agilent Technologies, USA), including a quaternary pump, an autosampler, a column temperature controller, and a diode array detector, connected to Agilent ChemStation software. The two chromatographic columns employed were an ACE EXCEL C_18_ column (250 × 4.6 mm, 5 *μ*m) and a JADE-PAK C_8_ column (50 × 4.6 mm, 3.5 *μ*m). The mobile phase was comprised of 0.1% aqueous phosphoric acid (*A*) and acetonitrile (B). The elution condition was set as follows: 5%–5% B for 0–5 min, 5%–14% B for 5–15 min, 14%–18% B for 15–21 min, 18%–21% B for 21–30 min, 21%–24% B for 30–35 min, 24%–30% B for 35–40 min, 30%–36% B for 40–50 min, 36%–95% B for 50–60 min, 95%–95% B for 60–65 min, and 95%–5% B for 65–75 min. A low rate of 0.6 mL/min and a temperature of 25°C were maintained. A wavelength used to detect signal was selected as 230 nm and the injection volume was set as 10 *μ*L.

### 2.4. Preparation of Reference Standard Solution and Sample Solution

Five kinds of reference substances were dissolved in methanol to prepare mixed reference standard solution, and the final concentration was as follows: calycosin 7-O-glucoside (0.00150 g/mL), syringin (0.00135 g/mL), icariin (0.00178 g/mL), chlorogenic acid (0.00294 g/mL), and hesperidin (0.00225 g/mL). One TMOL sample (10 mL) was taken from each batch for analysis. Methanol was added to the samples for ultrasonic extraction for 5 minutes; then the volume of the extract was adjusted to 25 mL. Furthermore, to remove the insoluble components, the centrifugation was performed for 10 min under the condition of 9668.16×g to obtain supernatant, which was filtered with 0.22 *μ*m membrane prior to injecting.

### 2.5. Data Analysis

“Similarity Evaluation System for Chromatographic Fingerprint of TCM” (Chinese Pharmacopoeia Commission, 2012A) was applied to analyze fingerprints. The similarity was calculated by the cosine of the angle between the sample fingerprinting and the reference fingerprinting with MATLAB (MathWorks, R2019a). The variability of HPLC fingerprint analysis of 12 batches of samples was evaluated by principal component analysis (PCA) with MATLAB (MathWorks, R2019a). Hierarchical clustering analysis was performed using Origin software (OriginLab, 2021b).

## 3. Results and Discussion

### 3.1. Optimization of Coupled Columns for Fingerprinting

Choices of chromatographic columns are based on key parameters such as stationary phase and lengths, which have a significant influence on selectivity and resolution. In this research, considering the relatively strong polarity of components of TMOL, the column with a shorter chain length has relatively greater polarity, and so reversed-phase chromatographic columns with different stationary phases and different chain lengths are combined for analysis. The optimal result of two connected columns was determined by trial and error according to the columns available in our research laboratory.

**Figure 2:**
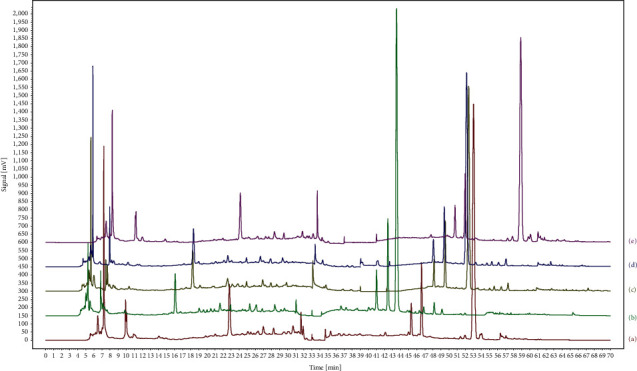
Separation results of different coupled columns by column switching technology. (a) Phenomenex Synergi Hydro-RP C18 (4 *μ*m, 250 × 4.6 mm) and JADE-PAK C8 (3.5 *μ*m, 50 × 4.6 mm), (b) ACE EXCEL C18 (5 *μ*m, 250 × 4.6 mm) and JADE-PAK C8 (3.5 *μ*m, 50 × 4.6 mm), (c) JADE-PAK C8 (5 *μ*m, 150 × 4.6 mm) and JADE-PAK ODS-AQ (5 *μ*m, 150 × 4.6 mm), (d) JADE-PAK C8 (5 *μ*m, 150 × 4.6 mm) and Sharpsil-U C4 (5 *μ*m, 150 × 4.6 mm), and (e) ACE EXCEL C18 (5 *μ*m, 250 × 4.6 mm) and JADE-PAK C8 (5 *μ*m, 150 × 4.6 mm).

With the purpose of obtaining more chromatographic peaks in the HPLC fingerprinting of TMOL, two columns with different stationary phases, such as Phenomenex Synergi Hydro-RP C_18_ (4 *μ*m, 250 × 4.6 mm) and JADE-PAK C_8_ (3.5 *μ*m, 50 × 4.6 mm), ACE EXCEL C_18_ (5 *μ*m, 250 × 4.6 mm) and JADE-PAK C_8_ (3.5 *μ*m, 50 × 4.6 mm), JADE-PAK C_8_ (5 *μ*m, 150 × 4.6 mm) and JADE-PAK ODS-AQ (5 *μ*m, 150 × 4.6 mm), and JADE-PAK C_8_ (5 *μ*m, 150 × 4.6 mm) and Sharpsil-U C_4_ (5 *μ*m, 150 × 4.6 mm), were connected through a six-way valve. In addition, the combination of ACE EXCEL C_18_ (5 *μ*m, 250 × 4.6 mm) and JADE-PAK C_8_ (5 *μ*m, 150 × 4.6 mm) was also tried; that is, the total column length is 400 mm, but the results showed the column pressure increased and the separation effect was not improved significantly. Therefore, we did not try further and the total length of the two columns was limited to 300 mm to prevent excessive column pressure. As shown in Figure 2, it is found that ACE EXCEL C_18_ (250 × 4.6 mm, 5 *μ*m) and JADE-PAK C_8_ (50 × 4.6 mm, 3.5 *μ*m) (combination b) could get the better peak capacity and resolution. A total of 56 peaks with peak heights greater than 10 mAU were separated by the two columns based on ACE EXCEL C_18_ (5 *μ*m, 250 × 4.6 mm) and JADE-PAK C_8_ (3.5 *μ*m, 50 × 4.6 mm), which was the best among the different column-connection modes, and the resolution of 34 peaks was more than 1.5. Through the first kind of connection method (shown in Figures 1(a) and 1(b)), more polar components can be separated by double-column analysis before 35 min. Through the second kind of connection method (shown in Figures 1(c) and 1(d)), more components with less polarity can be obtained by double-column analysis after 35 min.

### 3.2. Optimization of HPLC Chromatographic Conditions

The HPLC conditions including mobile phase with different acids, gradient elution, column temperature, flow rate, and detection wavelength were optimized for obtaining fingerprints with satisfactory separation. Acetonitrile was selected as a solvent eluent to obtain good chromatographic behavior and low background noise. Since the addition of acid additives in the mobile phase can reduce the tailing degree of the chromatographic peak and improve the peak shape, the effect of adding different acids (0.1% formic acid, 0.1% acetic acid, and 0.1% phosphoric acid) into mobile phase A was investigated, and the results showed that the effect of adding phosphoric acid was the best (Figure S1). By adjusting the proportion of the mobile phase, the baseline was stable, the retention time of each chromatographic peak was moderate, more peaks were obtained, and better separation was achieved. Different column temperatures (25°C, 30°C, and 35°C) were compared and the most appropriate column temperature was 25°C (Figure S2). According to rate theory, the flow rate can affect the plate height, and thus separation effect. Compared with different flow rates (0.5 mL/min, 0.6 mL/min, and 0.7 mL/min), the best condition was 0.6 mL/min with more peaks and better resolution (Figure S3). After testing different wavelengths (210 nm, 230 nm, 254 nm, 260 nm, and 270 nm), 230 nm was selected as the most appropriate detection wavelength because most compounds had satisfactory absorption (Figure S4).

### 3.3. Validation of the HPLC Fingerprint Method

**Figure 3:**
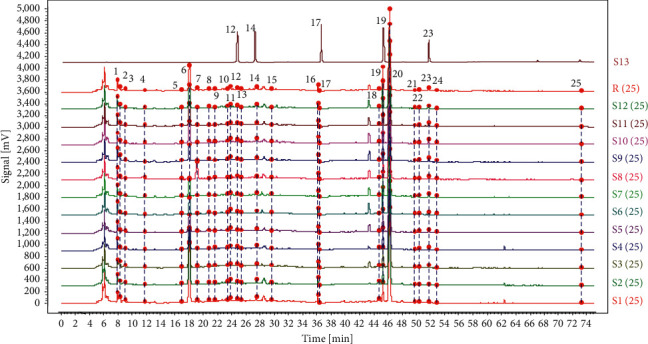
The fingerprints of 12 batches of TMOL, R reference fingerprint based on column switching technology, and (S13) the chromatogram of mixed reference standard solution. Peaks 12, 14, 17, 19, and 23 refer to syringin, chlorogenic acid, calycosin 7-O-glucoside, hesperidin, and icariin, respectively.

The characteristic peak of chlorogenic acid (peak 14 shown in Figure 3) was selected as a reference peak, and the relative standard deviations (RSDs, %) of relative retention time (RRT) and relative peak area (RPA) of the common peaks were used for validation of the HPLC fingerprint method, including the stability, repeatability, and intraday and interday precision. The specific results are displayed in Table S1. The stability test was performed by analysis of the same sample solution with different time intervals (0, 2, 4, 8, 12, and 24 h), and the RSDs values of RRT and RPA were less than 0.75% and 4.8%, respectively. The repeatability test was performed by analysis of six independent TMOL samples of the same batch, and the RSDs values of RRT and RPA were less than 0.72% and 4.8%, respectively. The intraday precision test was performed by analysis of the same solution six consecutive times within one day, and the RSDs values of RRT and RPA were less than 0.81% and 4.9%, respectively. The interday precision test was performed by analysis of the same solution three consecutive times over three consecutive days, and the RSDs values of RRT and RPA were less than 0.71% and 5.0%, respectively. The above results showed that the established method was feasible for the chromatographic fingerprint of TMOL.

### 3.4. Chromatographic Fingerprinting of TMOL

Under the above optimal chromatographic conditions, the fingerprints of 12 batches samples were obtained. According to their retention times (Figure 3) and ultraviolet (UV) spectra, five peaks are identified as syringin, chlorogenic acid, calycosin 7-O-glucoside, hesperidin, and icariin, respectively. The contrast of UV spectra for the above five components is shown in Figure S5.

The fingerprints of 12 batches of samples by column switching technology were input to professional software named Similarity Evaluation System for Chromatographic Fingerprint of TCM. A reference fingerprint obtained by the average method and the sample fingerprints are both shown in Figure 3. Because the column switching technology based on a single pump was used to establish the fingerprint method, the fingerprint measurement required two injections. The reference fingerprints and TMOL fingerprints of 12 batches of the first injection (before 35 min) and the second injection (after 35 min) are shown in Figures S6 and S7, respectively.

### 3.5. Similarity Analysis

Based on obtained common peaks, similarity analysis was performed between the reference fingerprint and the fingerprints of TMOL samples using MATLAB R2019a. The similarities were evaluated with the cosine method. The similarity of 10 unexpired samples were 0.999, 0.999, 0.999, 0.999, 0.998, 0.999, 0.997, 0.996, 0.996, and 0.998, and they shared similar chromatographic patterns with similarity values higher than 0.995. The two expired samples had lower similarity values, which were 0.978 and 0.984, suggesting the differences existed in quality between the expired and the unexpired TMOL.

The similarity threshold is the lowest limit of fingerprint similarity for a class of samples. Interval estimation is often used to determine the threshold in statistics, which requires that the overall distribution of the data is known or approximately empirical. The bootstrap method proposed by Professor Efron in 1979 is based on the existing small amount of sample data and extends to a large amount of sample data by replacement sampling [22]. Therefore, according to the results of similarity evaluation of 12 batches of samples, the bootstrap method was used to determine the identification threshold of the unexpired TMOL samples (the number of bootstrap samples to draw is 10000, *α* = 0.05). After calculation, the similarity threshold is 0.992. The results showed that the similarity of unexpired samples (S3-S12) was higher than 0.992, which could be considered similar and belong to the same category, while the similarity of expired samples (S1–S2) was lower than 0.992, which could be considered to be different from the quality of unexpired samples. The HPLC fingerprinting combined with similarity analysis and threshold determination could judge the quality discrepancy of TMOL samples.

### 3.6. Principal Component Analysis

PCA is a multivariate analysis technique widely used for feature extraction and dimensionality reduction to investigate the correlation between multiple variables [23]. The areas of 25 common peaks in chromatographic fingerprints as variables and 12 samples as objects were employed to form the data matrix, which was analyzed directly with PCA using MATLAB R2019a. Based on the eigenvalues and contribution rate of principal components, two principal components (PC1, PC2) were extracted to account for 87.1% and 10.9% of the 25 data variance in 12 batches of samples, respectively, and could well represent most information in the fingerprints of samples. The PCA score plot is shown in Figure S8. Twelve batches of TMOL samples distribute in two domains, which are labeled as group 1 (S1, S2) and group 2 (S3–S12), respectively. The boundary between the two categories is apparent. S1 and S2 are in positive part on PC1, while the rest of the samples (S3-S12) form an independent cluster with scores close to zero or negative values on PC1. On PC2, the scores of samples (S1, S2, S4, S7, and S11) are negative values, while other samples have positive scores.

Furthermore, to identify the variables causing the significant category separation, the loading plot for PC1 and PC2 is also shown in Figure S9. From the loading plot, peak 20 and peak 6 significantly contribute to the two categories. Peak 18 and peak 19 moderately lead to separation, while other peaks have little contribution to classification.

### 3.7. HCA with Heatmaps

**Figure 4:**
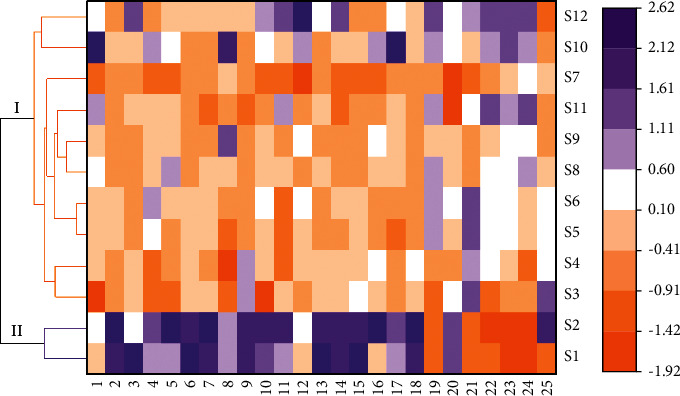
Hierarchical clustering analysis (HCA) with heatmaps based on the common peak areas for 12 batches of TMOL fingerprints.

In order to further clarify the influence of common peaks on classifiers, HCA with heatmaps was performed to group TMOL samples into different clusters. The peak areas of 12 batches of TMOL samples were normalized, and the cluster dendrograms were generated based on Euclidean distance with group average method for measuring the dissimilarity. Visibly, all TMOL samples were gathered into two clusters (Figure 4). Specifically, the expired samples 1 and 2 are located near to each other in one cluster, and the unexpired samples 3–10 are involved in another cluster. In addition, some peaks like 6, 20, 18, and 19 seem to have an obvious difference in peak area between the two clusters, which corresponds to the result obtained by the loading plot (Figure S9) of PCA. The results indicated that the fingerprints combined with HCA realized the identification of the expired and the unexpired TMOL samples.

### 3.8. Comparison with Fingerprinting Based on a Single Column

Three batches of TMOL samples (180101, 180104, and 180203) and the solution of the mixed standards were analyzed under the chromatographic conditions of the fingerprinting method based on a single column and column switching technology with one pump, respectively. HPLC fingerprints of 3 batches of TMOL samples and the chromatogram of mixed reference standard solution with the single-column method are shown in Figure S10. The peak capacities of the three batches of samples analyzed by the single-column method were 45, 46, and 46, while the corresponding peak capacities increased to 56, 63, and 62 by the column switching method, respectively. The column switching method could obtain a higher peak capacity than the single-column method (at least 11 chromatographic peaks) under the similar conditions. In addition, compared with the single-column method, the column switching method could increase the number of theoretical plates of the same component (Table S2) and achieve a better resolution of the five components (Table S3). Resolution (*R*) can be expressed as $R=\left(\sqrt{N}/4\right)\left(\alpha -1/\alpha \right)\left(k_{2}/k_{2}+1\right)$, and theoretical plate number (*N*), separation factor (*α*), and retention factor of the latter peak (*k*_2_) are three main parameters affecting resolution. Compared with the single column, the length of the column is increased by the column switching technology, which can acquire a higher theoretical plate number. Under the condition of the same mobile phase, the change of column stationary phase affects *α* and *k*_2_. Therefore, the improvement of resolution by column switching is attributed to the integrated effect of *N*, *α*, and *k*_2_.

As a comparison, there were 25 common peaks (Figure 3) in the fingerprints of different batches, including 16 peaks (Figure S6) in the first injection analysis and 9 peaks (Figure S7) in the second injection analysis, while 16 common peaks (Figure S11) were only obtained if we used a single C18 column for analysis [6] under similar measurement time. More common peaks occurred in the fingerprints, indicating more separation power with column switching technology than a single column, and could reflect chemical composition more comprehensively, which is highly meaningful for the quality evaluation of TMOL.

Traditional Chinese medicine (TCM) is different from chemically synthesized drugs in that its chemical composition is complex and the sources are extensive, making it difficult to control and evaluate its quality. HPLC fingerprinting technology is to separate the complicated chemical composition of TCM with chromatographic column under the optimal chromatographic conditions to acquire the corresponding chromatogram as fingerprint. The characteristic peaks of the fingerprint are the manifestation of the chemical composition of TCM, which can indicate its quality. Therefore, the fingerprint plays a key role in quality control and evaluation of TCM. The more peaks in fingerprint, the stronger characterization of TCM's quality. Although the column switch using a single pump due to laboratory conditions has the limited improvement on the HPLC fingerprinting separation, it would be still an alternative method to develop the fingerprint, which could have a certain positive point in quality evaluation of TCM.

## 4. Conclusions

A reliable fingerprinting method based on column switching technology by using a single pump was developed and applied successfully to analyze 12 batches of very complex preparation of TMOL in the present work. Through chemometric methods including similarity analysis, PCA, and HCA, the discrimination of expired and unexpired TMOL samples was successfully achieved. Column switching technology with a pump would make fingerprints have better separation power and higher peak capacity and could provide an effective method to establish fingerprints for quality control and evaluation of complex TCM preparations like TMOL.

## Data Availability

The data used to support the findings of this study are available from the corresponding author upon request.

## Supplementary Materials

Supplementary Materials The following materials are included in the supplementary materials. Figure S1: separation results of different acids as additives. Figure S2: separation results of different column temperatures. Figure S3: separation results of different flow rates. Figure S4: separation results of different detection wavelengths. Figure S5: contrast of UV spectra for (A) syringin, (B) calycosin 7-O-glucoside, (C) calycosin 7-O-glucoside, (D) hesperidin, and (E) icariin in (a) TMOL and (b) the mixed reference standard solution. Figure S6: the fingerprints of 12 batches of TMOL and the reference fingerprinting (the first injection analysis). Figure S7: the fingerprints of 12 batches of TMOL and the reference fingerprinting (the second injection analysis). Figure S8: PCA two-dimensional score plot of 12 batches of TMOL. The number refers to the sample number. Figure S9: two-dimensional loading plot of the 25 common fingerprinting peaks. The number refers to the peak number. Figure S10: the HPLC fingerprint of (a) 3 batches of TMOL samples and (b) the chromatogram of mixed reference standard solution based on the single-column method. Peaks 1–5 refer to syringin, chlorogenic acid, calycosin 7-O-glucoside, hesperidin, and icariin, respectively. Figure S11: the fingerprints of 12 batches of TMOL and reference fingerprint based on the single-column method. Table S1: the results of precision, repeatability, and stability tests of fingerprinting method validation. Table S2: the theoretical plate number (*N*) of the five components of two fingerprinting methods. Table S3: the resolutions (*R*) of the five components of two fingerprinting methods.
