# Supplementary material for: Chromatographic Fingerprinting Based on Column Switching Technology for Quality Evaluation of Tianmeng Oral Liquid
Source: Int J Anal Chem. 2021 Sep 29;2021:2514762. doi: 10.1155/2021/2514762 (PMC8494583; doi:10.1155/2021/2514762)
Supplement: Supplementary Materials — The following materials are included in the supplementary materials. Figure S1: separation results of different acids as additives. Figure S2: separation results of different column temperatures. Figure S3: separation results of different flow rates. Figure S4: separation results of different detection wavelengths. Figure S5: contrast of UV spectra for (A) syringin, (B) calycosin 7-O-glucoside, (C) calycosin 7-O-glucoside, (D) hesperidin, and (E) icariin in (a) TMOL and (b) the mixed reference standard solution. Figure S6: the fingerprints of 12 batches of TMOL and the reference fingerprinting (the first injection analysis). Figure S7: the fingerprints of 12 batches of TMOL and the reference fingerprinting (the second injection analysis). Figure S8: PCA two-dimensional score plot of 12 batches of TMOL. The number refers to the sample number. Figure S9: two-dimensional loading plot of the 25 common fingerprinting peaks. The number refers to the peak number. Figure S10: the HPLC fingerprint of (a) 3 batches of TMOL samples and (b) the chromatogram of mixed reference standard solution based on the single-column method. Peaks 1–5 refer to syringin, chlorogenic acid, calycosin 7-O-glucoside, hesperidin, and icariin, respectively. Figure S11: the fingerprints of 12 batches of TMOL and reference fingerprint based on the single-column method. Table S1: the results of precision, repeatability, and stability tests of fingerprinting method validation. Table S2: the theoretical plate number (N) of the five components of two fingerprinting methods. Table S3: the resolutions (R) of the five components of two fingerprinting methods. [file 2514762.f1.docx]

Supplementary material

Title: Chromatographic fingerprinting based on column switching technology for quality evaluation of Tianmeng oral liquid


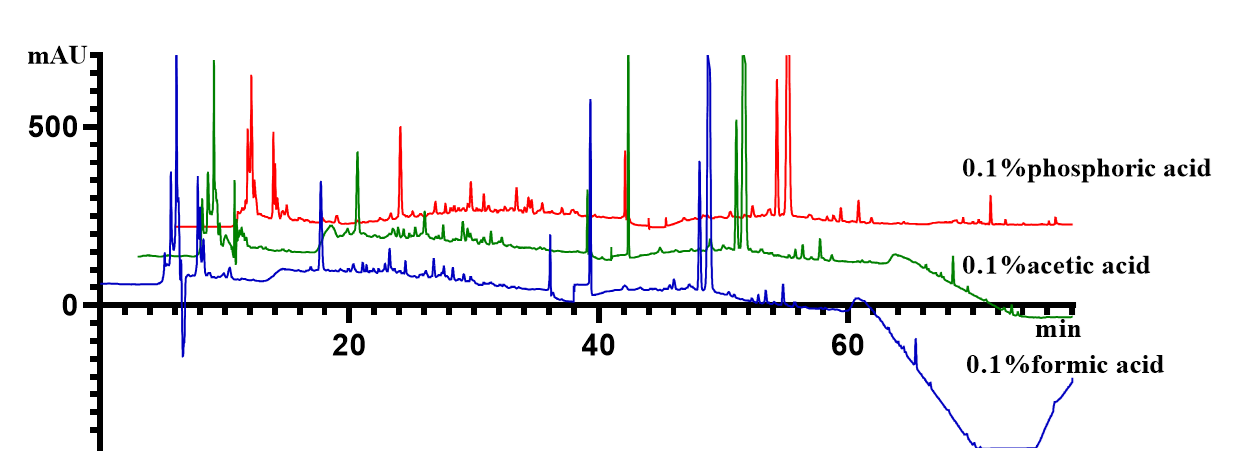


Figure S1: Separation results of different acids.


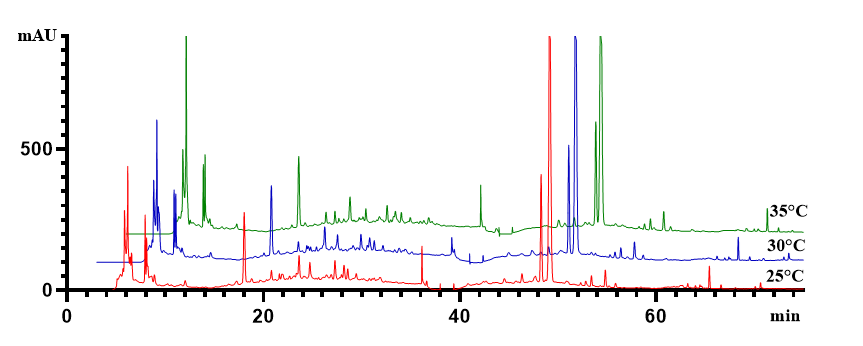


Figure S2: Separation results of different column temperatures.


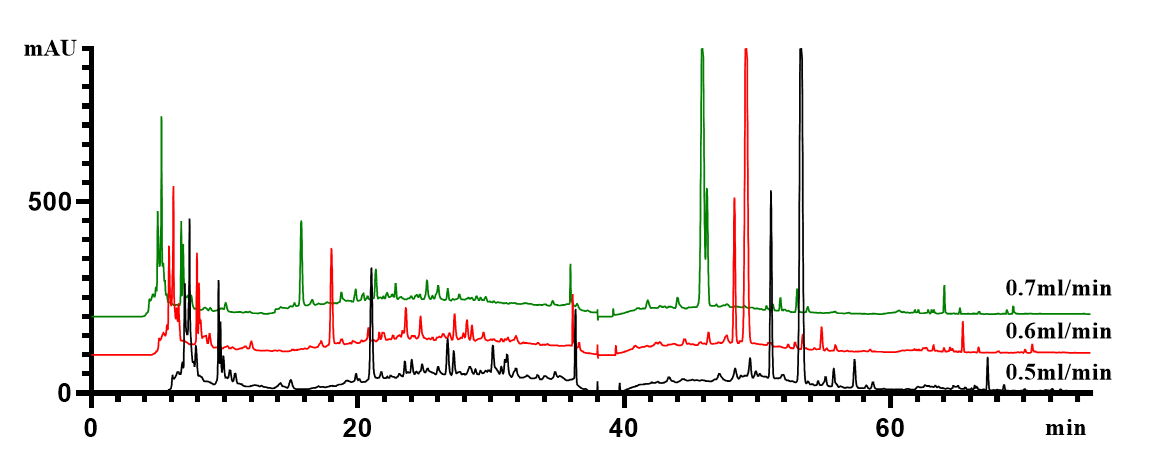


Figure S3: Separation results of different flow rates.

Figure S4: Separation results of different detection wavelengths.


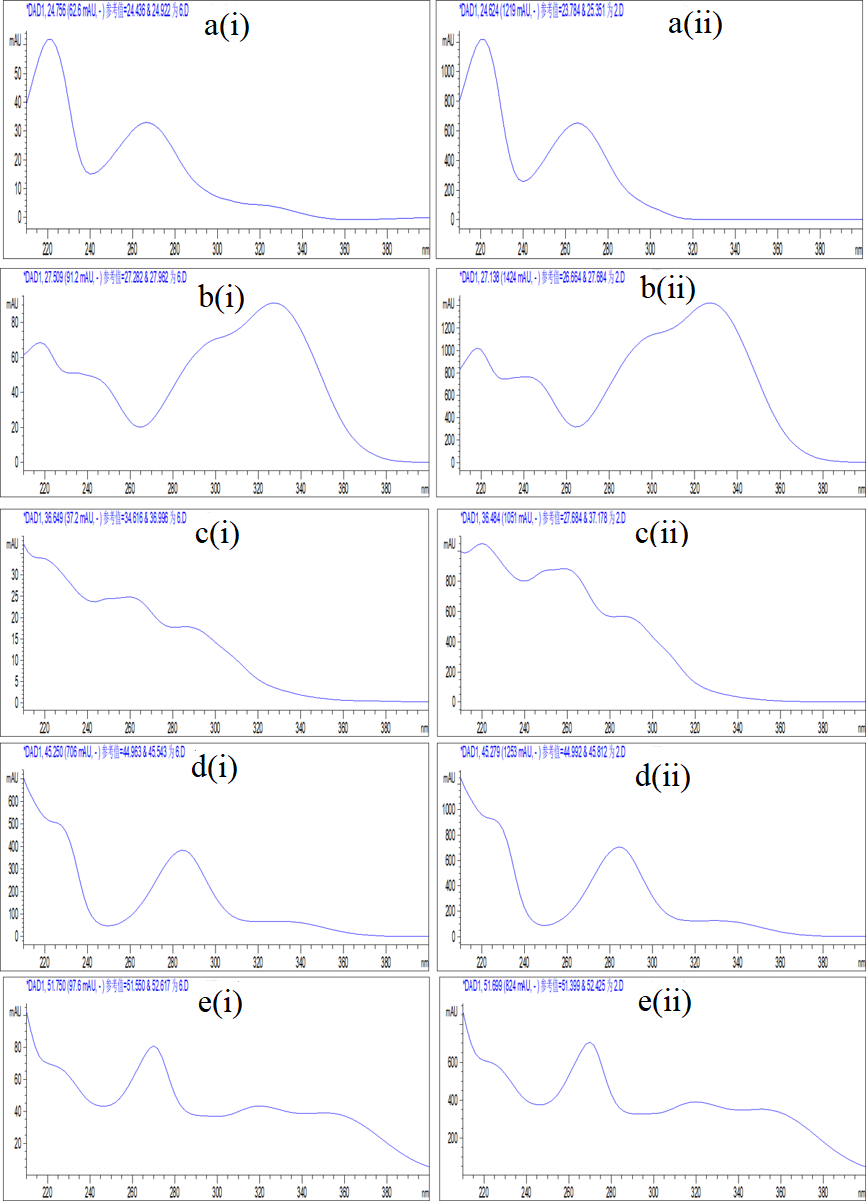


Figure S5: Contrast of UV spectra for (a) Syringin, (b) calycosin 7-O-glucoside, (c) calycosin 7-O-glucoside, (d) hesperidin, and (e) icariin in (i) TMOL and (ii)mixed reference standard solution.


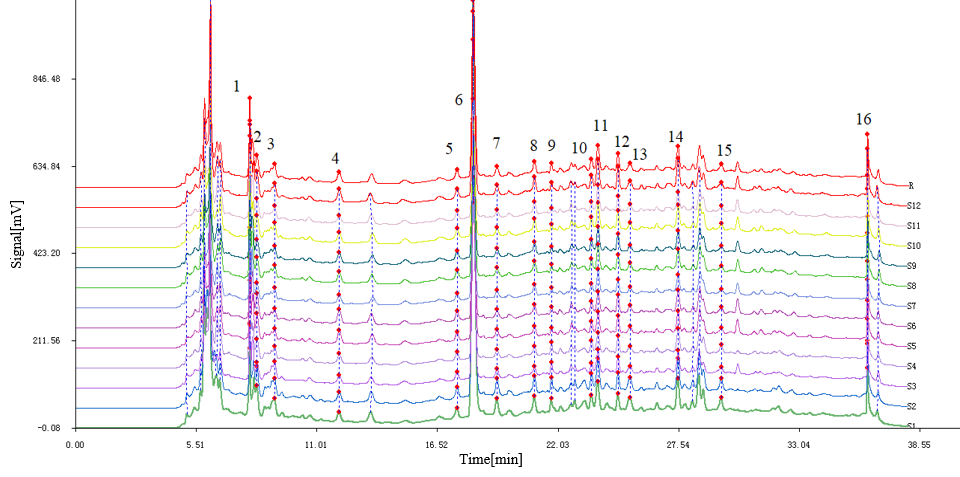


Figure S6: The fingerprints of 12 batches of TMOL and the reference fingerprinting (the first injection analysis).


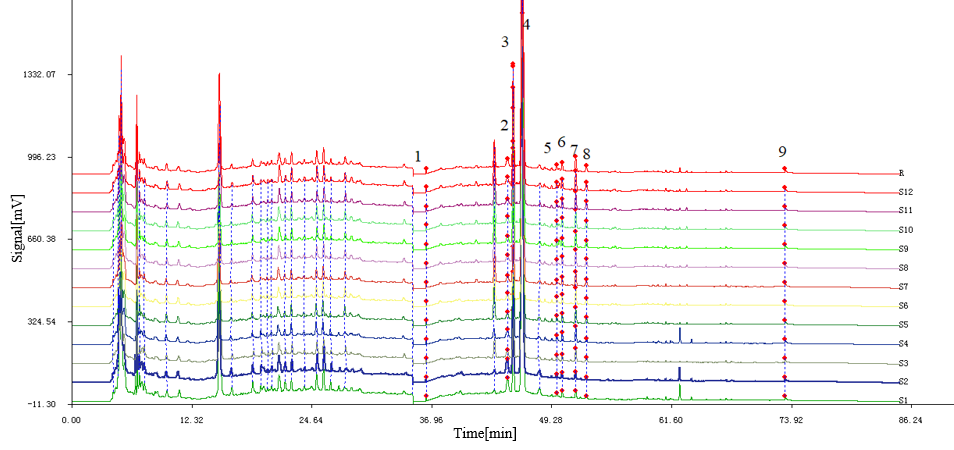


Figure S7: The fingerprints of 12 batches of TMOL and the reference fingerprinting (the second injection analysis).


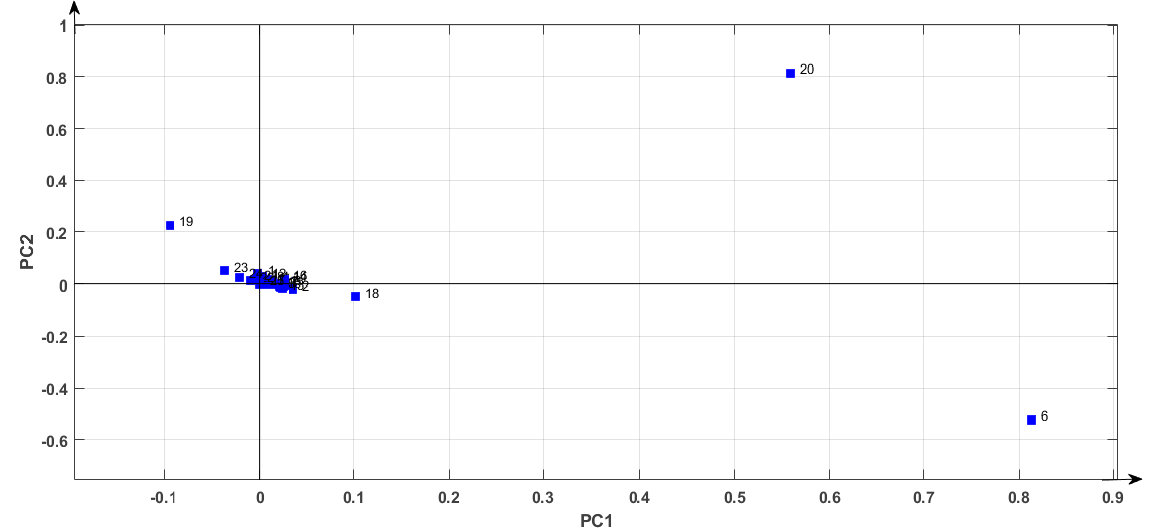

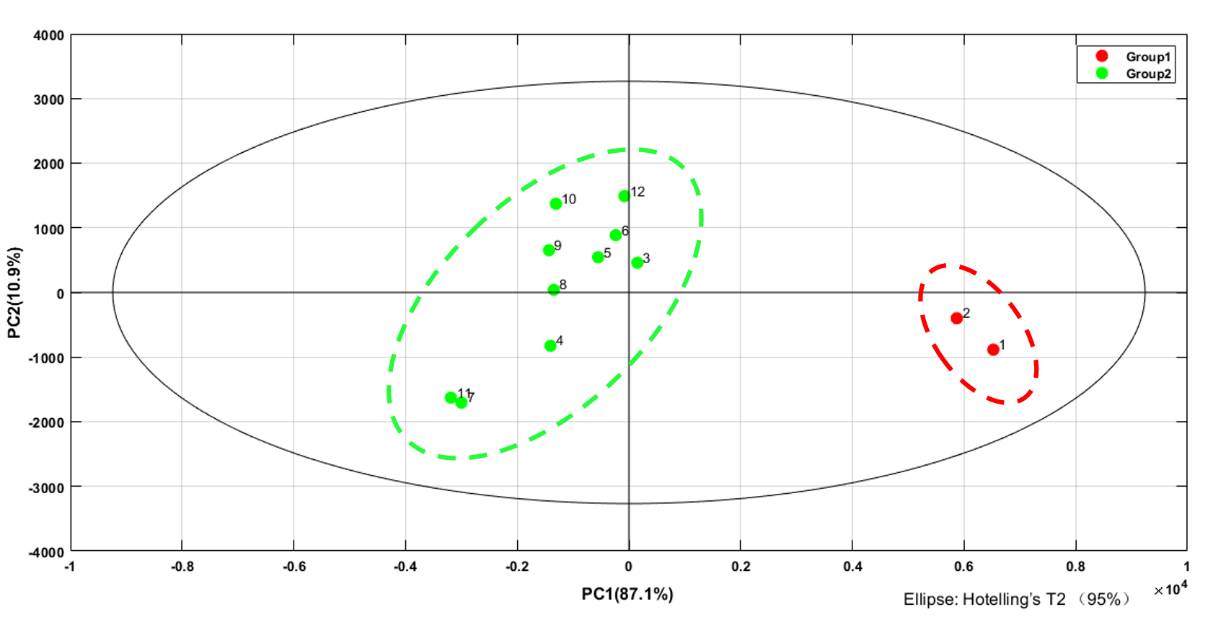
 Figure S8: PCA two-dimensional score plot of 12 batches of TMOL. The number refers to the sample number.

Figure S9: Two-dimensional loading plot of the 25 common fingerprinting peaks. The number refers to the peak number.


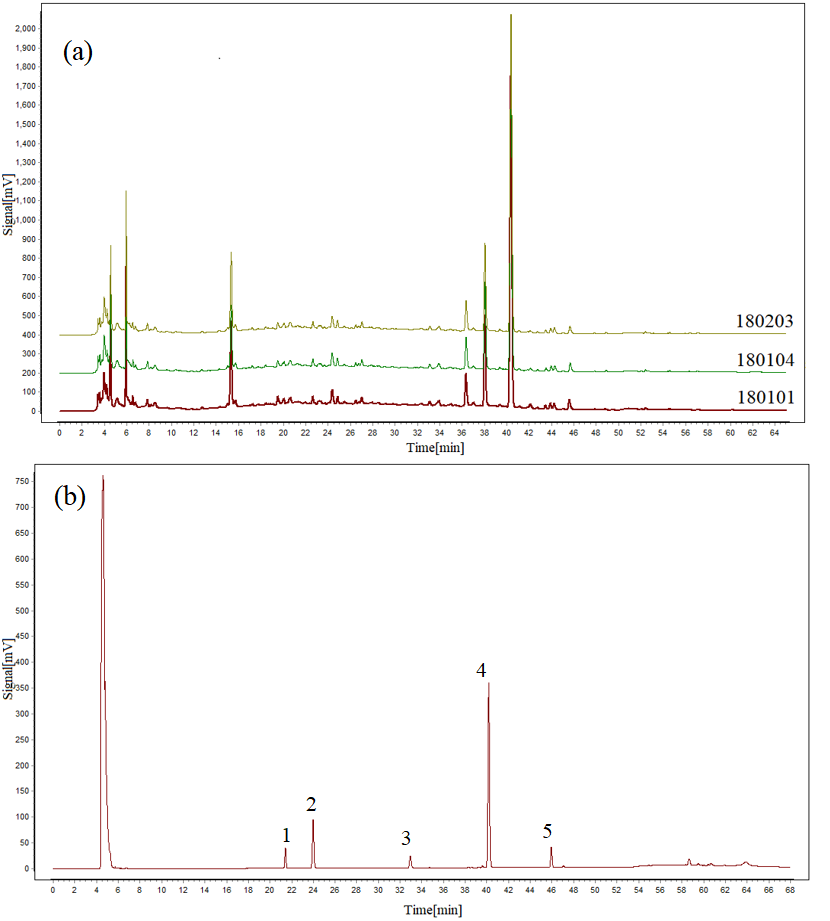


Figure S10: The HPLC fingerprint of (a) 3 batches of TMOL samples and (b) the chromatogram of mixed reference standard solution based on the single-column method. Peaks 1-5 refer to syringin, chlorogenic acid, calycosin 7-O-glucoside, hesperidin, and icariin, respectively.


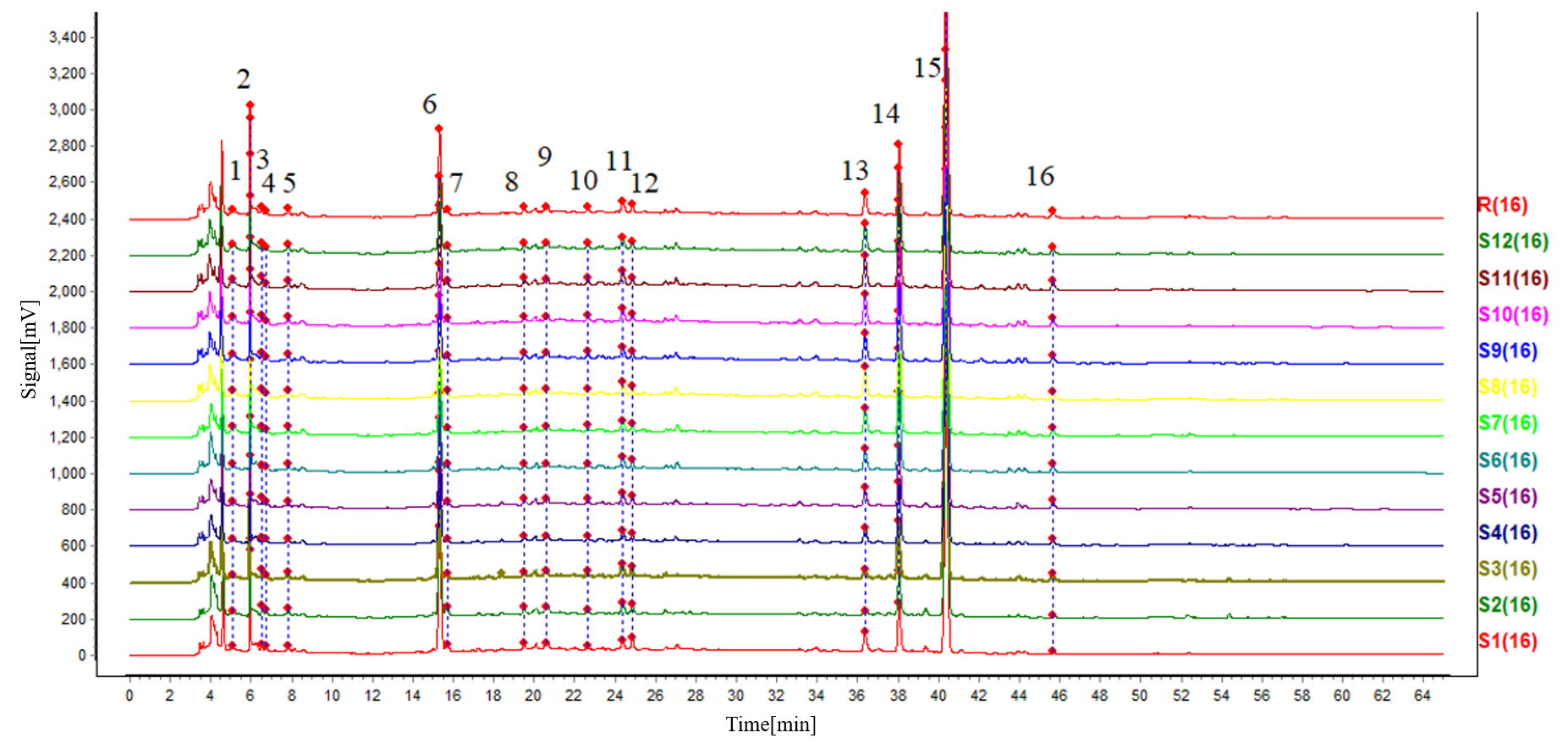
Figure S11: The fingerprints of 12 batches of TMOL samples and reference fingerprint based on the single-column method

Table S1: The results of precision, repeatability, and stability tests of fingerprinting method validation.

| Peak | Intra-day precision  (n = 6)  RSD (%) | | Inter-day precision (n = 9)  RSD (%) | | Stability  (24h, n = 6)  RSD (%) | | Repeatability  (n = 6)  RSD (%) | |
| --- | --- | --- | --- | --- | --- | --- | --- | --- |
|  | RPA | RRT | RPA | RRT | RPA | RRT | RPA | RRT |
| 1 | 1.7 | 0.30 | 3.2 | 0.34 | 1.8 | 0.29 | 2.8 | 0.41 |
| 2 | 4.9 | 0.58 | 4.5 | 0.71 | 3.5 | 0.54 | 2.7 | 0.72 |
| 3 | 3.3 | 0.57 | 4.8 | 0.55 | 3.0 | 0.47 | 3.7 | 0.46 |
| 4 | 3.5 | 0.45 | 3.6 | 0.51 | 2.0 | 0.40 | 2.8 | 0.35 |
| 5 | 4.2 | 0.47 | 2.6 | 0.32 | 3.1 | 0.46 | 3.0 | 0.32 |
| 6 | 1.7 | 0.32 | 3.2 | 0.23 | 1.0 | 0.30 | 1.8 | 0.22 |
| 7 | 4.2 | 0.27 | 4.3 | 0.28 | 3.3 | 0.25 | 3.8 | 0.22 |
| 8 | 4.8 | 0.16 | 2.8 | 0.15 | 4.7 | 0.14 | 4.0 | 0.18 |
| 9 | 4.3 | 0.11 | 3.0 | 0.13 | 4.5 | 0.08 | 1.1 | 0.13 |
| 10 | 4.6 | 0.81 | 4.6 | 0.11 | 4.8 | 0.75 | 2.3 | 0.19 |
| 11 | 2.5 | 0.04 | 4.7 | 0.28 | 2.4 | 0.03 | 1.4 | 0.08 |
| 12 | 1.1 | 0.06 | 4.1 | 0.11 | 1.9 | 0.09 | 1.8 | 0.05 |
| 13 | 2.5 | 0.11 | 3.8 | 0.20 | 2.0 | 0.11 | 4.8 | 0.10 |
| 14 | 0 | 0 | 0 | 0 | 0 | 0 | 0 | 0 |
| 15 | 3.5 | 0.05 | 4.9 | 0.16 | 3.7 | 0.04 | 3.4 | 0.15 |
| 16 | 4.1 | 0.35 | 5.0 | 0.11 | 4.8 | 0.37 | 2.8 | 0.29 |
| 17 | 2.9 | 0.29 | 4.7 | 0.10 | 2.9 | 0.34 | 4.3 | 0.30 |
| 18 | 3.4 | 0.55 | 2.8 | 0.22 | 2.2 | 0.56 | 3.7 | 0.44 |
| 19 | 2.1 | 0.43 | 4.0 | 0.23 | 1.5 | 0.34 | 1.8 | 0.46 |
| 20 | 1.2 | 0.44 | 4.5 | 0.22 | 1.6 | 0.38 | 1.8 | 0.46 |
| 21 | 1.7 | 0.36 | 4.2 | 0.45 | 1.5 | 0.40 | 2.7 | 0.39 |
| 22 | 1.9 | 0.34 | 3.3 | 0.23 | 2.6 | 0.25 | 2.0 | 0.40 |
| 23 | 1.5 | 0.37 | 2.4 | 0.23 | 1.4 | 0.26 | 2.0 | 0.41 |
| 24 | 2.2 | 0.40 | 4.2 | 0.24 | 3.4 | 0.38 | 3.0 | 0.55 |
| 25 | 4.9 | 0.27 | 3.9 | 0.10 | 4.8 | 0.36 | 4.5 | 0.30 |

Table S2: The theoretical plate number (*N*) of the five components of two fingerprinting methods.

| Method | *N* _Syringin_ | *N* _chlorogenic acid_ | *N* _calycosin 7-O-glucoside_ | *N* _hesperidin_ | *N* _icariin_ |
| --- | --- | --- | --- | --- | --- |
| Single column | 155077 | 126248 | 201882 | 267171 | 392187 |
| Column switching | 198109 | 188482 | 568697 | 504824 | 741898 |

Table S3: The resolutions (*R*) of the five components of two fingerprinting methods.

| Method | *R* _Syringin - chlorogenic acid_ | *R* _chlorogenic acid -calycosin 7-O-glucoside_ | *R* _calycosin 7-O-glucoside - hesperidin_ | *R* _hesperidin - icariin_ |
| --- | --- | --- | --- | --- |
| Single column | 6.24 | 28.88 | 16.48 | 23.97 |
| Column switching | 10.65 | 42.11 | 37.28 | 25.8 |
